# Hidden metabolic obesity phenotypes and risk of incident type 2 diabetes among BMI-defined non-obese adults: a secondary analysis of the NAGALA cohort

**DOI:** 10.3389/fendo.2026.1959132

**Published:** 2026-09-25

**Authors:** Chenrong Cai, Yang Wang

**Affiliations:** College of Traditional Chinese Medicine, Fujian University of Traditional Chinese Medicine, Fuzhou, Fujian, China

**Keywords:** abdominal obesity, body mass index, cohort study, fatty liver, hidden metabolic obesity, non-obese adults, type 2 diabetes mellitus

## Abstract

**Background:**

Body mass index (BMI) may fail to capture clinically relevant heterogeneity in adiposity-related diabetes risk, particularly among Asian adults who are not classified as obese. We investigated whether abdominal obesity and fatty liver identify distinct risks of incident type 2 diabetes mellitus (T2DM) among adults with BMI <25 kg/m^2^.

**Methods:**

This secondary analysis used data from the Japanese NAGALA cohort. A total of 12,940 adults without diabetes at baseline and with BMI <25 kg/m^2^ were classified into four phenotypes: neither abdominal obesity nor fatty liver, isolated abdominal obesity, isolated fatty liver, and combined abdominal obesity and fatty liver. Cox proportional hazards models were used to estimate hazard ratios (HRs) for incident T2DM. We additionally assessed adiposity-component burden, multiplicative and additive interaction, sex-specific associations, multiple sensitivity analyses, and incremental 5-year predictive performance.

**Results:**

During 78,694.5 person-years of follow-up, 201 incident T2DM events occurred. Incidence rates were 1.70, 2.61, 8.20, and 21.37 per 1,000 person-years across the four phenotypes, respectively. In the fully adjusted model, isolated abdominal obesity was not significantly associated with T2DM (HR 1.47, 95% CI 0.66–3.31), whereas isolated fatty liver (HR 3.43, 95% CI 2.41–4.87) and combined abdominal obesity and fatty liver (HR 8.10, 95% CI 4.65–14.12) were strongly associated with higher risk. Risk increased progressively with the number of non-BMI adiposity components (P for trend <0.001). Associations with fatty liver were observed in both sexes, with no clear evidence of effect modification by sex (P for interaction=0.176), and remained robust in sensitivity analyses, including restriction to BMI <23 kg/m^2^. Adding abdominal obesity, fatty liver, or the four-level phenotype to a comprehensive clinical model did not clearly improve 5-year predictive performance.

**Conclusions:**

Among BMI-defined non-obese adults, fatty liver—especially when accompanied by abdominal obesity—identified substantial heterogeneity in incident T2DM risk. These findings highlight the limitations of BMI alone for characterizing adiposity-related metabolic risk, while not establishing incremental predictive utility beyond established clinical risk factors.

## Graphical Abstract

**Figure:**
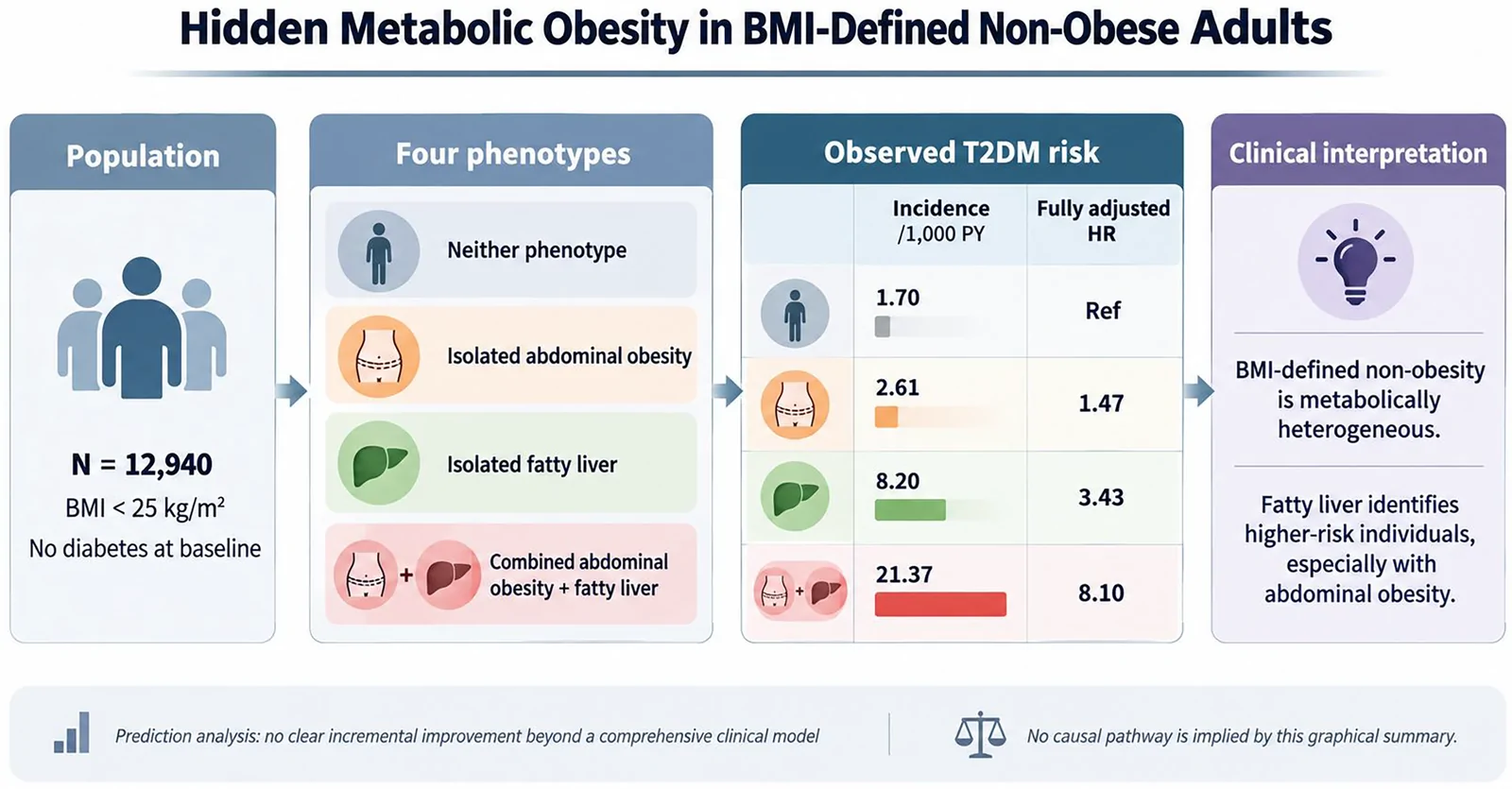
Study population, phenotype definitions, observed gradient in incident T2DM risk, and clinical implications. The graphical summary is descriptive and does not imply causality.

## 1 Introduction

Type 2 diabetes mellitus (T2DM) remains a major global health challenge because of its rapidly increasing prevalence (1, 2) and substantial burden of cardiovascular, renal, and microvascular complications (3–5). Although strategies for diabetes prevention have advanced (6, 7), a considerable proportion of individuals who eventually develop T2DM are not identified as high-risk under current screening frameworks (8, 9). This highlights the need for more refined risk stratification approaches that can detect susceptible individuals at earlier and potentially reversible stages of metabolic dysfunction (10).

Obesity is a well-established risk factor for T2DM (11), and body mass index (BMI) remains the most widely used measure of obesity in clinical practice and epidemiological research (12). However, BMI is an imperfect surrogate for adiposity because it does not adequately reflect fat distribution, visceral adiposity, or ectopic fat accumulation (12). This limitation is particularly relevant in Asian populations, among whom metabolic abnormalities and diabetes may occur even in individuals who are not classified as obese by BMI (13–15). Accordingly, increasing attention has been paid to the concept of “metabolically obese normal-weight” or “hidden metabolic obesity,” which emphasizes that the absence of BMI-defined obesity does not necessarily indicate metabolic health (16–18).

Abdominal obesity and fatty liver represent two important manifestations of hidden metabolic obesity (16–21). Abdominal obesity mainly reflects excess visceral adiposity (21), whereas fatty liver is a key marker of ectopic fat deposition (19, 20). Both conditions are closely associated with insulin resistance, hepatic glucose overproduction, chronic low-grade inflammation, and lipotoxicity (19, 21–23), and previous studies have linked them to increased diabetes risk (24, 25). Importantly, Okamura et al. previously evaluated combinations of BMI-defined obesity, abdominal obesity, and ectopic fat obesity in the overall NAGALA cohort and demonstrated a particularly strong association between ectopic fat obesity and incident T2DM (26). However, the extent to which abdominal obesity and fatty liver phenotypes distinguish diabetes risk specifically among individuals who all fall below the conventional BMI obesity threshold remains incompletely characterized. Within this BMI-defined non-obese population, the graded risk associated with increasing non-BMI adiposity burden and the joint effects of abdominal obesity and fatty liver also warrant focused evaluation.

The present secondary analysis does not seek to re-establish the general association between fatty liver (termed “ectopic fat obesity” in the original NAGALA study) and T2DM. Instead, it addresses the narrower question of residual diabetes-risk heterogeneity among adults with BMI <25 kg/m^2^ who would all be classified as non-obese by the conventional BMI threshold. We examined the associations of four abdominal obesity/fatty liver phenotypes with incident T2DM, evaluated the graded risk associated with the accumulation of these non-BMI adiposity components, assessed multiplicative and additive interaction, examined sex-related heterogeneity, and tested the robustness of the findings at a more stringent BMI threshold. We also performed an exploratory statistical decomposition of the association among abdominal obesity, fatty liver, and incident T2DM and evaluated whether adding these phenotypes provided incremental 5-year predictive information beyond established clinical risk factors.

## 2 Methods

### 2.1 Study design and participants

This study was a secondary analysis of a publicly available longitudinal cohort derived from the Dryad database (Data from: Ectopic fat obesity presents the greatest risk for incident type 2 diabetes: a population-based longitudinal study; dataset DOI: 10.5061/dryad.8q0p192) (26). The original study was conducted in a Japanese population-based health screening cohort with repeated follow-up examinations (26). The Dryad dataset included 15,464 participants who were free of diabetes at baseline (26).

The present analysis was designed to investigate the association between hidden metabolic obesity phenotypes and incident type 2 diabetes among BMI-defined non-obese adults. Therefore, participants were eligible for the present study if they had a baseline body mass index (BMI) <25 kg/m^2^, available information on abdominal obesity status, fatty liver status, follow-up duration, and incident diabetes status. Of the 15,464 participants in the original dataset, 2,524 participants with baseline BMI ≥25 kg/m^2^ were excluded. No participants were excluded because of missing data on BMI, abdominal obesity, fatty liver status, follow-up time, or incident diabetes status. Thus, the final analytic sample included 12,940 BMI-defined non-obese participants.

### 2.2 Ethics statement

The original NAGALA study was approved by the Asahi University Hospital Ethics Committee (IRB No. 2018-09-01) and was conducted in accordance with the Declaration of Helsinki. The study used an opt-out sampling design. Participants undergoing health checkups at Asahi University Hospital were invited to participate, and those unwilling to participate were excluded. According to the original NAGALA study, informed consent was obtained from all participants. The present study was a secondary analysis of the publicly available NAGALA dataset and did not involve additional participant recruitment.

### 2.3 Assessment of hidden metabolic obesity phenotypes

The primary exposure was the hidden metabolic obesity phenotype defined by the combination of abdominal obesity and fatty liver (26).

Abdominal obesity was defined as waist circumference ≥90 cm in men and ≥80 cm in women (26). Fatty liver was defined by abdominal ultrasonography according to the criteria used in the original NAGALA study (19, 26). In the original NAGALA publication, ultrasonographically detected fatty liver was operationally termed “ectopic fat obesity.” Because ectopic fat is a broader concept that includes lipid accumulation in multiple non-adipose organs and tissues, we use “fatty liver” throughout the present manuscript when referring specifically to the measured hepatic phenotype; the term “ectopic fat obesity” is retained only when referring to the terminology of the original NAGALA study.

Based on these two components, participants were classified into four mutually exclusive phenotypes (26):

1. neither abdominal obesity nor fatty liver,
2. isolated abdominal obesity,
3. isolated fatty liver, and
4. combined abdominal obesity and fatty liver.

### 2.4 Incident type 2 diabetes

Incident type 2 diabetes mellitus (T2DM) was ascertained using the outcome criteria applied in the original NAGALA cohort and established diagnostic standards (26, 27). Participants were classified as having incident T2DM if they met any of the following criteria during follow-up: fasting plasma glucose ≥126 mg/dL (7.0 mmol/L), HbA1c ≥6.5% (48 mmol/mol), or a self-reported diagnosis of diabetes (26, 27). Follow-up time was calculated from the baseline examination to the first diagnosis of T2DM or the last available follow-up examination, whichever occurred first. Participants who remained free of T2DM were censored at the date of their last follow-up examination.

### 2.5 Covariates

Potential confounders were selected according to prior knowledge, clinical relevance, and data availability. Demographic factors included age and sex. Lifestyle factors included smoking status, alcohol consumption, and habitual exercise. Clinical covariates included systolic blood pressure, diastolic blood pressure, and baseline BMI (11, 12). Lipid parameters, liver enzymes, fasting plasma glucose, and HbA1c were not included in the primary models because they may represent intermediate metabolic factors or variables closely related to diabetes development (27). To assess whether the observed associations were independent of baseline glycemic status, fasting plasma glucose and HbA1c were additionally included in a sensitivity analysis (27).

### 2.6 Statistical analysis

Baseline characteristics were summarized according to hidden metabolic obesity phenotypes. Continuous variables were presented as mean ± standard deviation or median with interquartile range, as appropriate, whereas categorical variables were summarized as frequencies and percentages. Differences across phenotype groups were compared using analysis of variance, Kruskal–Wallis tests, chi-square tests, or Fisher’s exact tests, as appropriate.

Follow-up time was calculated from baseline to the occurrence of incident type 2 diabetes or the last follow-up examination. Incidence rates were calculated using person-time at risk according to the following formula: Incidence rate (per 1,000 person-years) = (number of incident T2DM cases/total person-years at risk) × 1,000 (38). Total person-years at risk were calculated as the sum of each participant’s follow-up time from baseline until the first diagnosis of T2DM or the last available follow-up examination, whichever occurred first. Cox proportional hazards regression models were used to estimate hazard ratios (HRs) and 95% confidence intervals (CIs) for the association between hidden metabolic obesity phenotypes and incident type 2 diabetes. Three sequential models were constructed. Model 1 was adjusted for age and sex. Model 2 was further adjusted for smoking status, alcohol consumption, and habitual exercise. Model 3 was additionally adjusted for systolic blood pressure, diastolic blood pressure, and baseline BMI and was considered the primary fully adjusted model. Sex-specific analyses were conducted by fitting Models 1–3 separately in men and women, omitting sex from the covariates. Overall interactions between sex and the four-level phenotype were assessed in the pooled sample using three-degree-of-freedom likelihood-ratio tests comparing models with and without three interaction terms between sex and the phenotype indicators.

An exploratory mediation analysis was performed as a statistical decomposition of the association among abdominal obesity, fatty liver, and incident type 2 diabetes. The analysis was conducted using the mediation package in R, with abdominal obesity as the exposure, fatty liver as the putative mediator, and incident type 2 diabetes as the outcome. The model-based indirect effect, direct effect, total effect, and proportion mediated were estimated. Because abdominal obesity and fatty liver were measured concurrently at baseline, their temporal sequence could not be established. Accordingly, this analysis was considered exploratory and hypothesis-generating and was not interpreted as evidence of a causal mediation pathway.

Incremental predictive performance was evaluated at 5 years (1826.25 days). The clinical base Cox model included age, sex, continuous BMI, systolic and diastolic blood pressure, smoking status, alcohol consumption, habitual exercise, fasting plasma glucose, and HbA1c. Smoking status and alcohol consumption were modeled as categorical variables. Three extended models additionally included abdominal obesity, fatty liver, or the four-level phenotype. Predictive performance was assessed using the cumulative/dynamic time-dependent area under the receiver operating characteristic curve (AUC), Brier score, continuous net reclassification improvement (NRI), and absolute integrated discrimination improvement (IDI), accounting for censoring using inverse probability of censoring weighting. Confidence intervals for changes in predictive metrics were estimated using 500 paired participant-level bootstrap resamples, with all models refitted within each resample. Bootstrap optimism-corrected AUC and Brier score estimates were additionally calculated to assess potential overfitting.

Several sensitivity analyses were conducted to assess the robustness of the findings. These included additional adjustment for fasting plasma glucose and HbA1c, exclusion of participants who developed diabetes within the first year of follow-up, exclusion of heavy alcohol drinkers, restriction to participants with BMI <23 kg/m^2^, and exclusion of participants with baseline diabetic-range glycemic markers, defined as fasting plasma glucose ≥126 mg/dL or HbA1c ≥6.5% (27).

All statistical analyses were performed using R software version 4.5.1. Cox proportional hazards regression analyses were conducted using the survival package, and mediation analyses were performed using the mediation package. All statistical tests were two-sided, and a P value <0.05 was considered statistically significant.

## 3 Results

### 3.1 Baseline characteristics according to hidden metabolic obesity phenotypes

The baseline characteristics of the study participants are presented in **Table 1**. Among 12,940 BMI-defined non-obese participants included in the primary analysis, 11,102 had neither abdominal obesity nor fatty liver, 501 had isolated abdominal obesity, 1,196 had isolated fatty liver, and 141 had combined abdominal obesity and fatty liver. Baseline characteristics differed significantly across the four phenotypes. Participants with combined abdominal obesity and fatty liver were older than those with neither phenotype (49.93 ± 7.80 vs. 43.22 ± 9.00 years; P<0.001). Sex distribution also differed markedly across groups (P<0.001): isolated abdominal obesity was predominantly observed in women (90.4%), whereas isolated fatty liver was predominantly observed in men (87.2%). During follow-up, 201 incident type 2 diabetes events were documented among BMI-defined non-obese participants. The proportion of incident diabetes increased across hidden metabolic obesity phenotypes, from 1.0% among participants with neither abdominal obesity nor fatty liver to 1.4% in those with isolated abdominal obesity, 5.1% in those with isolated fatty liver, and 12.8% in those with combined abdominal obesity and fatty liver.

**Table 1:** Baseline characteristics of BMI-defined non-obese participants according to hidden metabolic obesity phenotypes.

Characteristic	Overall (n=12940)	Neither abdominal obesity nor fatty liver (n=11102)	Isolated abdominal obesity (n=501)	Isolated fatty liver (n=1196)	Combined abdominal obesity and fatty liver (n=141)	*P*
Age, years	43.64 ± 8.99	43.22 ± 9.00	47.04 ± 8.98	45.36 ± 8.26	49.93 ± 7.80	<0.001
Sex (%)						<0.001
Women	6406 (49.5)	5716 (51.5)	453 (90.4)	153 (12.8)	84 (59.6)	
Men	6534 (50.5)	5386 (48.5)	48 (9.6)	1043 (87.2)	57 (40.4)	
Smoking status (%)						<0.001
Never	7846 (60.6)	6843 (61.6)	405 (80.8)	505 (42.2)	93 (66.0)	
Past	2349 (18.2)	1927 (17.4)	50 (10.0)	347 (29.0)	25 (17.7)	
Current	2745 (21.2)	2332 (21.0)	46 (9.2)	344 (28.8)	23 (16.3)	
Alcohol consumption (%)						<0.001
None/low	9955 (76.9)	8518 (76.7)	427 (85.2)	901 (75.3)	109 (77.3)	
Light	1467 (11.3)	1279 (11.5)	47 (9.4)	125 (10.5)	16 (11.3)	
Moderate	1104 (8.5)	955 (8.6)	22 (4.4)	113 (9.4)	14 (9.9)	
Heavy	414 (3.2)	350 (3.2)	5 (1.0)	57 (4.8)	2 (1.4)	
Exercise habit (%)						<0.001
No	10614 (82.0)	9045 (81.5)	434 (86.6)	1014 (84.8)	121 (85.8)	
Yes	2326 (18.0)	2057 (18.5)	67 (13.4)	182 (15.2)	20 (14.2)	
BMI, kg/m^2^	21.11 ± 2.14	20.79 ± 2.06	23.10 ± 1.27	22.99 ± 1.42	23.93 ± 0.93	<0.001
Waist circumference, cm	74.03 ± 7.32	72.73 ± 6.83	83.45 ± 3.51	80.60 ± 4.84	86.77 ± 4.88	<0.001
Body weight, kg	57.52 ± 9.09	56.44 ± 8.88	60.35 ± 6.79	65.44 ± 7.21	65.21 ± 8.42	<0.001
Systolic blood pressure, mmHg	112.35 ± 14.07	111.30 ± 13.75	115.63 ± 14.57	119.78 ± 14.01	120.81 ± 13.84	<0.001
Diastolic blood pressure, mmHg	70.12 ± 9.94	69.40 ± 9.74	71.37 ± 10.05	75.58 ± 9.81	76.04 ± 9.19	<0.001
Fasting plasma glucose, mg/dL	92.21 ± 7.35	91.65 ± 7.25	92.19 ± 6.87	96.84 ± 6.76	96.56 ± 6.41	<0.001
HbA1c, %	5.15 ± 0.31	5.14 ± 0.31	5.25 ± 0.32	5.25 ± 0.33	5.39 ± 0.30	<0.001
Total cholesterol, mg/dL	196.21 ± 32.94	194.38 ± 32.56	203.19 ± 33.52	208.46 ± 32.93	211.23 ± 31.69	<0.001
HDL cholesterol, mg/dL	58.45 ± 15.57	59.72 ± 15.49	58.60 ± 14.22	47.53 ± 12.35	50.96 ± 12.56	<0.001
Triglycerides, mg/dL	60.00 (41.00-89.00)	57.00 (39.00-82.00)	60.00 (43.00-88.00)	103.00 (74.00-149.00)	92.00 (67.00-125.00)	<0.001
ALT, IU/L	16.00 (12.00-21.00)	15.00 (12.00-20.00)	15.00 (12.00-18.00)	25.00 (19.00-34.00)	22.00 (16.00-29.00)	<0.001
AST, IU/L	17.00 (14.00-20.00)	17.00 (14.00-20.00)	17.00 (14.00-20.00)	19.00 (16.00-24.00)	19.00 (16.00-22.00)	<0.001
GGT, IU/L	14.00 (11.00-20.00)	14.00 (11.00-19.00)	13.00 (11.00-17.00)	22.00 (16.00-32.00)	19.00 (15.00-27.00)	<0.001
Follow-up, years	5.52 (2.78-9.49)	5.59 (2.79-9.56)	4.87 (2.90-7.89)	5.79 (2.73-9.98)	5.64 (2.32-9.13)	<0.001
Incident diabetes (%)						<0.001
No	12739 (98.4)	10987 (99.0)	494 (98.6)	1135 (94.9)	123 (87.2)	
Yes	201 (1.6)	115 (1.0)	7 (1.4)	61 (5.1)	18 (12.8)	

Data are presented as n (%), mean +/- standard deviation, or median (interquartile range). P values were obtained using the Kruskal-Wallis test for continuous variables and the chi-square test for categorical variables, as appropriate.

ALT, alanine aminotransferase; AST, aspartate aminotransferase; BMI, body mass index; DBP, diastolic blood pressure; GGT, gamma-glutamyl transferase; HbA1c, glycated hemoglobin; HDL, high-density lipoprotein; SBP, systolic blood pressure.

Despite all participants having BMI <25 kg/m^2^, participants with hidden metabolic obesity showed higher adiposity and a more unfavorable metabolic profile. Mean BMI and waist circumference were highest in the combined abdominal obesity and fatty liver group compared with the reference group (BMI: 23.93 ± 0.93 vs. 20.79 ± 2.06 kg/m^2^; waist circumference: 86.77 ± 4.88 vs. 72.73 ± 6.83 cm; both P<0.001). Fatty liver-related phenotypes showed particularly adverse glucose, lipid, and liver enzyme profiles. Compared with participants with neither phenotype, those with isolated fatty liver had higher fasting plasma glucose (96.84 ± 6.76 vs. 91.65 ± 7.25 mg/dL), higher triglycerides [103.00 (74.00–149.00) vs. 57.00 (39.00–82.00) mg/dL], lower HDL cholesterol (47.53 ± 12.35 vs. 59.72 ± 15.49 mg/dL), and higher ALT [25.00 (19.00–34.00) vs. 15.00 (12.00–20.00) IU/L], all with P<0.001. HbA1c was highest in the combined phenotype group (5.39 ± 0.30% vs. 5.14 ± 0.31% in the reference group; P<0.001). These findings suggest that abdominal and especially ectopic fat accumulation identified metabolically unfavorable phenotypes that were not captured by BMI status alone.

### 3.2 Incidence rates of type 2 diabetes according to hidden metabolic obesity phenotypes

The phenotype-specific incidence rates are summarized graphically in **Figure 1**. The incidence rate was lowest in participants with neither abdominal obesity nor fatty liver and increased markedly in those with isolated fatty liver and the combined phenotype. The proportional hazards assumption was further assessed using Schoenfeld residuals; the global test did not indicate a major overall violation of the proportional hazards assumption (P>0.05).

**Figure 1:**
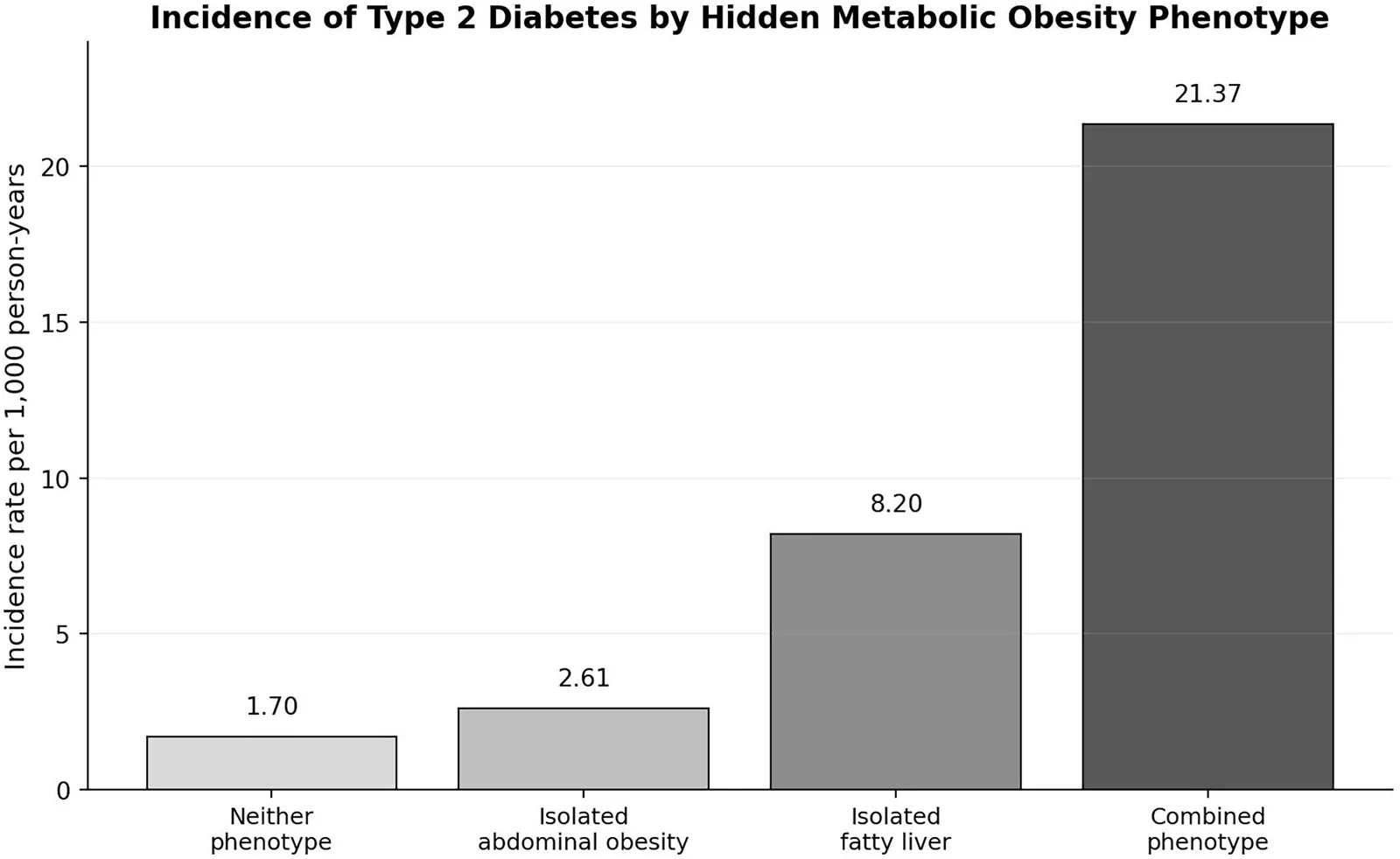
Incidence rates of type 2 diabetes according to hidden metabolic obesity phenotypes. Incidence rates are expressed per 1,000 person-years. T2DM, type 2 diabetes mellitus.

**Table 2** shows the incidence rates of incident type 2 diabetes according to hidden metabolic obesity phenotypes. During 78,694.5 person-years of follow-up, 201 incident diabetes events were documented among BMI-defined non-obese participants. The incidence rate was lowest among participants with neither abdominal obesity nor fatty liver, at 1.70 per 1,000 person-years, and was only slightly higher in those with isolated abdominal obesity, at 2.61 per 1,000 person-years. In contrast, participants with isolated fatty liver had a markedly higher incidence rate of 8.20 per 1,000 person-years, whereas the highest incidence rate was observed in those with combined abdominal obesity and fatty liver, at 21.37 per 1,000 person-years.

**Table 2:** Incidence rates of type 2 diabetes according to hidden metabolic obesity phenotypes.

Hidden metabolic obesity phenotype	Participants	Events	Person-years	Incidence rate per 1000 person-years
Neither abdominal obesity nor fatty liver	11102	115	67733.7	1.70
Isolated abdominal obesity	501	7	2679.6	2.61
Isolated fatty liver	1196	61	7438.7	8.20
Combined abdominal obesity and fatty liver	141	18	842.5	21.37

Incidence rate (per 1,000 person-years) = (number of incident T2DM cases/total person-years at risk) × 1,000. Total person-years at risk were calculated as the sum of individual follow-up times from baseline until incident T2DM or the last available follow-up examination, whichever occurred first (38).

T2DM, type 2 diabetes mellitus.

### 3.3 Associations of hidden metabolic obesity phenotypes with incident type 2 diabetes

**Table 3** shows the associations between hidden metabolic obesity phenotypes and incident type 2 diabetes. In Model 1 adjusted for age and sex, isolated abdominal obesity was associated with a higher but non-significant risk of incident diabetes compared with participants with neither abdominal obesity nor fatty liver [HR 1.78, 95% CI 0.82–3.89; P = 0.147]. In contrast, isolated fatty liver was significantly associated with a higher risk of incident diabetes [HR 3.76, 95% CI 2.72–5.19; P<0.001], and the strongest association was observed for combined abdominal obesity and fatty liver [HR 9.11, 95% CI 5.51–15.08; P<0.001]. After further adjustment for smoking status, alcohol consumption, and exercise habit in Model 2, the results remained largely unchanged: isolated abdominal obesity remained non-significant [HR 1.72, 95% CI 0.79–3.76; P = 0.173], whereas isolated fatty liver [HR 3.85, 95% CI 2.78–5.34; P<0.001] and combined abdominal obesity and fatty liver [HR 9.75, 95% CI 5.88–16.17; P<0.001] remained strongly associated with incident diabetes. In the fully adjusted Model 3, which additionally adjusted for systolic blood pressure, diastolic blood pressure, and baseline BMI, the association for isolated abdominal obesity was further attenuated and remained statistically non-significant [HR 1.47, 95% CI 0.66–3.31; P = 0.347]. By contrast, isolated fatty liver remained associated with a more than three-fold higher risk of incident diabetes [HR 3.43, 95% CI 2.41–4.87; P<0.001]. The highest risk was consistently observed among participants with combined abdominal obesity and fatty liver, with an approximately eight-fold higher risk of incident diabetes [HR 8.10, 95% CI 4.65–14.12; P<0.001].

**Table 3:** Associations of hidden metabolic obesity phenotypes with incident type 2 diabetes.

Phenotype	Model 1 HR (95% CI)	P	Model 2 HR (95% CI)	P	Model 3 HR (95% CI)	P
Neither abdominal obesity nor fatty liver	1.00 (Ref.)	–	1.00 (Ref.)	–	1.00 (Ref.)	–
Isolated abdominal obesity	1.78 (0.82-3.89)	0.147	1.72 (0.79-3.76)	0.173	1.47 (0.66-3.31)	0.347
Isolated fatty liver	3.76 (2.72-5.19)	<0.001	3.85 (2.78-5.34)	<0.001	3.43 (2.41-4.87)	<0.001
Combined abdominal obesity and fatty liver	9.11 (5.51-15.08)	<0.001	9.75 (5.88-16.17)	<0.001	8.10 (4.65-14.12)	<0.001

Hazard ratios and 95% confidence intervals were estimated using Cox proportional hazards regression models. Model 1 was adjusted for age and sex. Model 2 was further adjusted for smoking status, alcohol consumption, and exercise habit. Model 3 was further adjusted for systolic blood pressure, diastolic blood pressure, and baseline BMI. The group with neither abdominal obesity nor fatty liver was used as the reference.

BMI, body mass index; CI, confidence interval; HR, hazard ratio; Ref, reference.

Sex-specific analyses showed that isolated fatty liver and combined abdominal obesity and fatty liver were associated with incident T2DM in both men and women (**Supplementary Table S2**). In Model 3, the HRs for isolated fatty liver were 3.32 (95% CI, 2.27–4.86) in men and 4.57 (95% CI, 1.87–11.16) in women. For combined abdominal obesity and fatty liver, the corresponding HRs were 5.61 (95% CI, 2.57–12.25) and 14.17 (95% CI, 5.90–34.04), respectively. Associations for isolated abdominal obesity were not statistically significant in either sex. Despite the higher point estimates in women, there was no clear evidence of effect modification by sex (overall P for interaction = 0.176). The interaction test allowing sex-specific baseline hazards yielded P = 0.141.

### 3.4 Number of hidden metabolic obesity components and incident type 2 diabetes

We further examined whether the number of hidden metabolic obesity components was associated with incident type 2 diabetes (**Table 4**). A clear graded association was observed. Compared with participants without abdominal obesity or fatty liver, those with one hidden metabolic obesity component had a significantly higher risk of incident diabetes in the fully adjusted model, with an HR of 3.03 (95% CI, 2.16–4.25; *P* < 0.001). Participants with both abdominal obesity and fatty liver had an even higher risk, with an HR of 8.42 (95% CI, 4.83–14.67; *P* < 0.001). The trend across increasing numbers of hidden metabolic obesity components was statistically significant (*P* for trend <0.001).

**Table 4:** Associations between the number of hidden metabolic obesity components and incident type 2 diabetes.

Score	Definition	Participants	Events	Model 1 HR (95% CI)	P	Model 2 HR (95% CI)	P	Model 3 HR (95% CI)	P
0	No abdominal obesity or fatty liver	11102	115	1.00 (Ref.)	–	1.00 (Ref.)	–	1.00 (Ref.)	–
1	One hidden metabolic obesity component	1697	68	3.34 (2.46-4.51)	<0.001	3.36 (2.48-4.56)	<0.001	3.03 (2.16-4.25)	<0.001
2	Two hidden metabolic obesity components	141	18	9.16 (5.54-15.16)	<0.001	9.83 (5.93-16.30)	<0.001	8.42 (4.83-14.67)	<0.001
*P* for trend								<0.001	

The hidden metabolic obesity score was calculated as abdominal obesity plus fatty liver and ranged from 0 to 2. The P for trend was estimated by modeling the score as an ordinal variable in the fully adjusted model.

BMI, body mass index; CI, confidence interval; HR, hazard ratio; Ref, reference.

### 3.5 Interaction between abdominal obesity and fatty liver

**Table 5** summarizes the multiplicative and additive interaction analyses between abdominal obesity and fatty liver in relation to incident type 2 diabetes. In the fully adjusted model, no statistically significant multiplicative interaction was observed between abdominal obesity and fatty liver (P for interaction=0.323). On the additive scale, the RERI was 4.20 (95% CI, −0.04 to 8.44), the attributable proportion due to interaction was 0.52 (95% CI, 0.23–0.80), and the synergy index was 2.45 (95% CI, 0.67–4.23). Point estimates suggested a possible positive additive interaction. However, the 95% confidence interval for the RERI included 0 and that for the synergy index included 1, indicating substantial statistical uncertainty.

**Table 5:** Interaction between abdominal obesity and fatty liver on incident type 2 diabetes.

Scale	Term or metric	Estimate	95% CI	P
Multiplicative	Abdominal obesity	1.47	0.66-3.31	0.347
Multiplicative	Fatty liver	3.43	2.41-4.87	<0.001
Multiplicative	Combined abdominal obesity and fatty liver	8.10	4.65-14.12	<0.001
Multiplicative	Abdominal obesity x fatty liver			0.323
Additive	RERI	4.20	-0.04-8.44	
Additive	AP	0.52	0.23-0.80	
Additive	SI	2.45	0.67-4.23	

Multiplicative interaction was assessed by adding a product term between abdominal obesity and fatty liver to the fully adjusted Cox model. Additive interaction was evaluated using relative excess risk due to interaction, attributable proportion due to interaction, and synergy index.

AP, attributable proportion due to interaction; CI, confidence interval; RERI, relative excess risk due to interaction; SI, synergy index.

### 3.6 Exploratory mediation analysis

**Table 6** presents the results of the exploratory mediation analysis examining the statistical decomposition of the association among abdominal obesity, fatty liver, and incident type 2 diabetes among BMI-defined non-obese participants. The estimated indirect effect was 0.0032 (95% CI, 0.0016–0.0054; P<0.001), the estimated direct effect was 0.0155 (95% CI, 0.0042–0.0313; P = 0.002), and the total effect was 0.0187 (95% CI, 0.0066–0.0357; P<0.001). The model-based indirect effect corresponded to approximately 17.6% (95% CI, 8.8%–40.1%) of the estimated total association. Because abdominal obesity and fatty liver were assessed at the same baseline examination, these estimates should not be interpreted as demonstrating that fatty liver temporally or causally mediates the association between abdominal obesity and incident T2DM.

**Table 6:** Mediation analysis of fatty liver in the association between abdominal obesity and incident type 2 diabetes among BMI-defined non-obese participants.

Effect	Estimate	95% CI lower	95% CI upper	*P* value
Model-based indirect effect (ACME)	0.003210542	0.00160404	0.005433892	<0.001
Model-based direct effect (ADE)	0.015467877	0.004166809	0.031283286	0.002
Total effect	0.018678419	0.006604017	0.035736704	<0.001
Proportion mediated	0.175639719	0.087763578	0.40072192	<0.001

Mediation analysis was performed using the R package mediation. Abdominal obesity was treated as the exposure, fatty liver as the putative mediator, and incident type 2 diabetes as the outcome. Estimates are presented on the risk-difference scale. The model was adjusted for age, sex, smoking status, alcohol consumption, exercise habit, systolic blood pressure, diastolic blood pressure, and baseline BMI. ACME and ADE are the conventional estimands returned by the mediation package; because abdominal obesity and fatty liver were measured concurrently at baseline, they are interpreted here as model-based indirect and direct effects rather than as evidence of causal mediation.

### 3.7 Incremental predictive performance

At 5 years, the clinical base model had an apparent AUC of 0.8984, compared with 0.8979 after adding abdominal obesity, 0.9051 after adding fatty liver, and 0.9046 after adding the four-level phenotype (**Supplementary Table S3**). For fatty liver, the change in AUC was +0.0067 (95% CI, −0.0004 to +0.0181), the continuous NRI was +0.1378 (95% CI, −0.1818 to +0.4276), and the absolute IDI was +0.0061 (95% CI, −0.0036 to +0.0198). For the four-level phenotype, the corresponding estimates were +0.0062 (95% CI, −0.0007 to +0.0171), +0.0389 (95% CI, −0.2319 to +0.4804), and +0.0033 (95% CI, −0.0068 to +0.0194), respectively. Brier scores were essentially unchanged across the models. Bootstrap optimism-corrected AUCs were 0.8932 for the base model, 0.8927 after adding abdominal obesity, 0.8997 after adding fatty liver, and 0.8992 after adding the four-level phenotype. The confidence intervals for the change in AUC, continuous NRI, and absolute IDI included zero for all three model extensions, providing no clear evidence of incremental predictive improvement beyond the clinical base model.

### 3.8 Sensitivity analyses

**Supplementary Table S1** summarizes the results of sensitivity analyses evaluating the robustness of the associations between hidden metabolic obesity phenotypes and incident type 2 diabetes. After further adjustment for fasting plasma glucose and HbA1c, the associations were attenuated but remained statistically significant for isolated fatty liver and combined abdominal obesity and fatty liver. In this conservative model, isolated fatty liver was associated with an HR of 2.09 (95% CI, 1.47–2.97; P<0.001), and combined abdominal obesity and fatty liver was associated with an HR of 4.25 (95% CI, 2.46–7.34; P<0.001). Isolated abdominal obesity remained non-significant.

The main findings were also robust after excluding participants who developed diabetes within the first year of follow-up and after excluding heavy alcohol drinkers. In these analyses, isolated fatty liver remained associated with approximately 3.5-fold higher diabetes risk, and combined abdominal obesity and fatty liver remained associated with more than eight-fold higher risk. When the analysis was restricted to participants with BMI <23 kg/m^2^, isolated fatty liver remained significantly associated with incident diabetes (HR 3.91, 95% CI, 2.48–6.15; P<0.001), and the combined phenotype also remained strongly associated with risk (HR 12.90, 95% CI, 4.53–36.72; P<0.001). In this restricted analysis, the isolated abdominal obesity group had no diabetes events and was therefore not estimable.

## 4 Discussion

In this large-scale secondary analysis of a Japanese health screening cohort, hidden metabolic obesity phenotypes were strongly associated with incident type 2 diabetes mellitus (T2DM) among adults with BMI <25 kg/m^2^. Fatty liver showed a stronger and more consistent association with diabetes risk than abdominal obesity alone, while individuals with both abdominal obesity and fatty liver had the highest risk. Diabetes risk also increased progressively with the accumulation of metabolic fat components. Sex-specific analyses showed that fatty liver and the combined phenotype were associated with incident T2DM in both men and women, although there was no clear statistical evidence of effect modification by sex. In contrast, the additional prediction analyses did not provide clear evidence that abdominal obesity, fatty liver, or the four-level phenotype materially improved 5-year individual-level risk prediction beyond a comprehensive clinical base model. Together, these findings demonstrate substantial adiposity-related risk heterogeneity within BMI-defined non-obesity while distinguishing risk association from incremental predictive performance.

The present findings should be interpreted as an extension of, rather than an independent replication of, the original NAGALA analysis. The association between fatty liver (termed “ectopic fat obesity” in the original NAGALA analysis) and incident T2DM was previously demonstrated by Okamura et al. (26); therefore, this association itself should not be regarded as novel. The principal incremental contribution of the present study is the focused characterization of heterogeneity in adiposity-related diabetes risk among individuals uniformly classified as non-obese by BMI, together with analyses of cumulative metabolic fat burden, multiplicative and additive interaction, sex-related heterogeneity, robustness at BMI <23 kg/m^2^, and incremental predictive performance.

The present findings have clinical and public health implications, but they should be interpreted in light of the distinction between risk association and prediction. Reliance on BMI alone may overlook substantial adiposity-related risk heterogeneity, particularly in Asian populations in whom visceral adiposity, ectopic fat accumulation, and metabolic abnormalities may occur at relatively low BMI levels (12–15, 21). The strong association between fatty liver and incident T2DM suggests that hepatic fat accumulation is a clinically relevant risk marker among BMI-defined non-obese individuals (25, 28, 29). However, our additional prediction analyses did not establish that incorporating abdominal obesity, fatty liver, or the four-level phenotype improves individual-level 5-year diabetes prediction beyond a clinical model that already includes BMI, glycemic markers, blood pressure, and lifestyle factors. Thus, the present results should not be interpreted as evidence for routine incorporation of these phenotypes into diabetes prediction algorithms. Dedicated prediction studies with external validation, calibration assessment, and evaluation of clinical utility are needed to determine whether these measures add actionable information to established risk assessment approaches.

Several biological mechanisms may explain the observed associations. Fatty liver reflects ectopic fat accumulation in the liver and is closely related to hepatic insulin resistance, increased hepatic gluconeogenesis, impaired insulin clearance, and systemic metabolic dysregulation (19, 20, 33, 34). These abnormalities can promote chronic hyperinsulinemia, fasting hyperglycemia, and progressive deterioration of glucose metabolism (35). Abdominal obesity, on the other hand, reflects excess visceral adiposity (12, 21, 24). Visceral fat is metabolically active and can increase free fatty acid flux to the liver through the portal circulation, thereby promoting hepatic lipid accumulation, inflammation, and insulin resistance (21, 33–35). The coexistence of abdominal obesity and fatty liver may therefore represent a state of intensified metabolic overload involving both visceral and hepatic fat compartments (21, 33, 34). This may partly explain why individuals with combined phenotypes had the highest risk of incident T2DM. Overall, these findings are consistent with the concept that ectopic and visceral fat accumulation, rather than total body size alone, plays a central role in the pathogenesis of T2DM (12, 21, 24, 33–35).

In the interaction analyses, we did not observe a statistically significant multiplicative interaction between abdominal obesity and fatty liver. Additive interaction measures suggested a possible combined effect, although the confidence intervals indicated substantial statistical uncertainty (36). These findings should therefore not be interpreted as definitive evidence of biological synergy. The exploratory mediation analysis yielded a model-based indirect-effect estimate corresponding to approximately 17.6% of the total association (37). Although this statistical decomposition is compatible with an indirect pathway involving fatty liver, abdominal obesity and fatty liver were measured concurrently at baseline, precluding establishment of temporal ordering. Reverse or bidirectional relationships between visceral and hepatic fat, as well as shared upstream determinants, cannot be excluded. Accordingly, the mediation findings should be regarded solely as exploratory and hypothesis-generating rather than as evidence of a causal mechanism.

### 4.1 Strengths and limitations

This study has several strengths. First, the longitudinal design allowed us to evaluate the temporal association between baseline hidden metabolic obesity phenotypes and incident T2DM. Second, the study included a large health screening cohort and focused specifically on BMI-defined non-obese adults, a population in whom diabetes risk may be underestimated (12–15). Third, the simultaneous assessment of abdominal obesity and fatty liver enabled a more refined characterization of hidden metabolic obesity phenotypes (16–21). Fourth, the consistency of the association results across multiple sensitivity analyses, including additional adjustment for glycemic markers, exclusion of early events, and restriction to BMI <23 kg/m^2^, supports their robustness. Finally, the additional sex-specific, interaction, and predictive-performance analyses allowed us to address heterogeneity and to distinguish strong epidemiological associations from demonstrated improvements in individual risk prediction.

Several limitations should also be acknowledged. First, as a secondary analysis of observational data, residual confounding cannot be fully excluded despite multivariable adjustment. Second, fatty liver was assessed by ultrasonography rather than quantitative imaging methods such as magnetic resonance imaging or computed tomography, which may have introduced exposure misclassification (19, 30). Third, detailed information on diet, genetic susceptibility, physical activity intensity, and medication use was limited, which may have influenced the observed associations. Fourth, several sex–phenotype subgroups contained relatively few incident T2DM events, resulting in wide confidence intervals; therefore, the absence of a statistically significant sex interaction should not be interpreted as establishing equivalent associations between men and women. Fifth, although we evaluated several measures of incremental predictive performance and used bootstrap optimism correction, these prediction analyses were conducted within the same cohort, and the changes in AUC, NRI, and IDI were imprecisely estimated. External validation is therefore required before any predictive application can be considered. Sixth, abdominal obesity and fatty liver were assessed only at baseline; therefore, changes in visceral adiposity, hepatic steatosis, or metabolic status during follow-up could not be evaluated. Finally, abdominal obesity and fatty liver were measured concurrently, so temporal ordering between the exposure and putative mediator cannot be established. The mediation analysis was therefore exploratory and should not be interpreted as establishing a causal mechanism (37).

## 5 Conclusion

In conclusion, among adults with BMI <25 kg/m^2^, hidden metabolic obesity phenotypes—particularly fatty liver alone or in combination with abdominal obesity—were strongly associated with an increased risk of incident T2DM. These findings demonstrate substantial heterogeneity in diabetes risk among individuals who would all be classified as non-obese according to BMI and underscore the limitations of using BMI alone to characterize adiposity-related metabolic risk. However, the present study did not establish that incorporating these phenotypes improves individual-level diabetes prediction beyond established clinical risk factors. Future studies should validate these findings in diverse populations, determine the predictive and clinical utility of non-BMI adiposity measures, and evaluate whether interventions targeting hepatic fat, visceral adiposity, or both can reduce T2DM risk in BMI-defined non-obese individuals (30–33).

## Data Availability

Publicly available datasets were analyzed in this study. This data can be found here: https://datadryad.org/stash/dataset/doi:10.5061/dryad.8q0p192.

## Supplementary material

Supplementary Table 1 Sensitivity analyses.

Supplementary Table 2 Sex-specific associations of hidden metabolic obesity phenotypes with incident type 2 diabetes.

Supplementary Table 3 Incremental predictive performance for 5-year incident type 2 diabetes.
