# Supplementary material for: Hidden metabolic obesity phenotypes and risk of incident type 2 diabetes among BMI-defined non-obese adults: a secondary analysis of the NAGALA cohort
Source: Front Endocrinol (Lausanne). 2026 Sep 25;17:1959132. doi: 10.3389/fendo.2026.1959132 (PMC13649437; doi:10.3389/fendo.2026.1959132)
Supplement: Supplementary file 1 [file SupplementaryFile1.pdf]

Supplementary Table 1. Sensitivity analyses.

Sensitivity analysis	Phenotype	N	Events	Model 3 HR (95% CI)	P
Further adjusted for FPG and HbA1c	Isolated abdominal obesity	12940	201	1.41 (0.62-3.19)	0.407
Further adjusted for FPG and HbA1c	Isolated fatty liver	12940	201	2.09 (1.47-2.97)	<0.001
Further adjusted for FPG and HbA1c	Combined abdominal obesity and fatty liver	12940	201	4.25 (2.46-7.34)	<0.001
Exclude diabetes within first follow-up year	Isolated abdominal obesity	12929	190	1.64 (0.73-3.69)	0.236
Exclude diabetes within first follow-up year	Isolated fatty liver	12929	190	3.59 (2.50-5.16)	<0.001
Exclude diabetes within first follow-up year	Combined abdominal obesity and fatty liver	12929	190	9.08 (5.18-15.93)	<0.001
Exclude heavy alcohol drinkers	Isolated abdominal obesity	12526	182	1.61 (0.71-3.63)	0.256
Exclude heavy alcohol drinkers	Isolated fatty liver	12526	182	3.54 (2.45-5.12)	<0.001
Exclude heavy alcohol drinkers	Combined abdominal obesity and fatty liver	12526	182	8.57 (4.87-15.07)	<0.001
Restrict to BMI <23 kg/m2	Isolated abdominal obesity	10064	125	Not estimable	
Restrict to BMI <23 kg/m2	Isolated fatty liver	10064	125	3.91 (2.48-6.15)	<0.001
Restrict to BMI <23 kg/m2	Combined abdominal obesity and fatty liver	10064	125	12.90 (4.53-36.72)	<0.001
Exclude baseline diabetic-range glycemic markers	Isolated abdominal obesity	12940	201	1.47 (0.66-3.31)	0.347
Exclude baseline diabetic-range glycemic markers	Isolated fatty liver	12940	201	3.43 (2.41-4.87)	<0.001
Exclude baseline diabetic-range glycemic markers	Combined abdominal obesity and fatty liver	12940	201	8.10 (4.65-14.12)	<0.001

Note: Sensitivity analyses were based on the fully adjusted model. In the BMI <23 kg/m2 analysis, the isolated abdominal obesity group had zero diabetes events and was therefore not estimable.

Abbreviations: BMI, body mass index; CI, confidence interval; HR, hazard ratio.

Supplementary Table 2. Sex-specific associations of hidden metabolic obesity phenotypes with incident type 2 diabetes.

Phenotype	N	Events	Model 1 HR (95% CI)	P	Model 2 HR (95% CI)	P	Model 3 HR (95% CI)	P
Men								
Neither abdominal obesity nor fatty liver	5386	82	1.00 (Ref.)	-	1.00 (Ref.)	-	1.00 (Ref.)	-
Isolated abdominal obesity	48	2	2.18 (0.53-8.87)	0.278	1.93 (0.47-7.90)	0.362	1.87 (0.45-7.82)	0.392
Isolated fatty liver	1043	54	3.36 (2.38-4.74)	<0.001	3.48 (2.45-4.93)	<0.001	3.32 (2.27-4.86)	<0.001
Combined abdominal obesity and fatty liver	57	8	5.45 (2.62-11.34)	<0.001	5.95 (2.85-12.43)	<0.001	5.61 (2.57-12.25)	<0.001
Women								
Neither abdominal obesity nor fatty liver	5716	33	1.00 (Ref.)	-	1.00 (Ref.)	-	1.00 (Ref.)	-
Isolated abdominal obesity	453	5	2.14 (0.82-5.59)	0.120	2.06 (0.78-5.44)	0.145	1.48 (0.52-4.17)	0.463
Isolated fatty liver	153	7	6.67 (2.92-15.25)	<0.001	6.44 (2.82-14.73)	<0.001	4.57 (1.87-11.16)	<0.001
Combined abdominal obesity and fatty liver	84	10	20.86 (9.96-43.68)	<0.001	21.99 (10.42-46.41)	<0.001	14.17 (5.90-34.04)	<0.001
P for sex interaction				0.077		0.122		0.176

Note: Cox models were fitted separately in men and women. Model 1 was adjusted for age. Model 2 additionally included smoking status, alcohol consumption (both categorical), and exercise habit. Model 3 additionally included systolic blood pressure, diastolic blood pressure, and baseline BMI. The group with neither abdominal obesity nor fatty liver was the reference within each sex.

Abbreviations: BMI, body mass index; CI, confidence interval; HR, hazard ratio; Ref., reference.

Supplementary Table 3. Incremental predictive performance for 5-year incident type 2 diabetes.

Model	5-year AUC (95% CI)	Change in AUC (95% CI)	Continuous NRI (95% CI)	Absolute IDI (95% CI)	5-year Brier score (95% CI)	P for LRT
Base model	0.8984 (0.8596-0.9380)	Ref.	Ref.	Ref.	0.006912 (0.005292-0.008137)	-
Base model + abdominal obesity	0.8979 (0.8582-0.9374)	-0.0005 (-0.0033 to +0.0017)	-0.0559 (-0.4231 to +0.3902)	-0.0023 (-0.0064 to +0.0023)	0.006935 (0.005327-0.008154)	0.002
Base model + fatty liver	0.9051 (0.8685-0.9418)	+0.0067 (-0.0004 to +0.0181)	+0.1378 (-0.1818 to +0.4276)	+0.0061 (-0.0036 to +0.0198)	0.006883 (0.005236-0.008123)	<0.001
Base model + four-level phenotype	0.9046 (0.8674-0.9418)	+0.0062 (-0.0007 to +0.0171)	+0.0389 (-0.2319 to +0.4804)	+0.0033 (-0.0068 to +0.0194)	0.006908 (0.005250-0.008113)	<0.001

Note: All table estimates are apparent (uncorrected), and changes, NRI, IDI, and likelihood-ratio tests compare each extended model with the base model. The base model included age, sex, BMI, systolic and diastolic blood pressure, smoking status, alcohol consumption, exercise habit, fasting plasma glucose, and HbA1c. The four-level phenotype added 3 degrees of freedom, equivalent to abdominal obesity, fatty liver, and their product term.

Abbreviations: AUC, area under the time-dependent receiver operating characteristic curve; BMI, body mass index; CI, confidence interval; HbA1c, glycated hemoglobin; IDI, integrated discrimination improvement; LRT, likelihood-ratio test; NRI, net reclassification improvement; Ref., reference. Methodological reference: doi:10.1002/sim.5647.
